# Conversion of waste cooking oil to environmentally acceptable surfactants for enhanced oil recovery

**DOI:** 10.1038/s41598-026-55003-z

**Published:** 2026-06-06

**Authors:** A. M. Al Sabagh, Fatma H. Abdel-Salam, Asmaa Mohamed

**Affiliations:** 1 Egyptian Petroleum Research Institute (EPRI), Nasr City, 11727 Cairo Egypt; 2 Chemistry Department, Faculty of Science (Girls), Al-Azhar University, Cairo, Egypt

**Keywords:** Ecofriendly surfactants, Waste cooking oil, phase behavior, Interfacial tension, Enhanced oil recovery, Chemistry, Energy science and technology, Engineering, Environmental sciences

## Abstract

Enhanced oil recovery (EOR) is a tertiary method used to extract crude oil remaining in reservoirs. With growing concerns about waste generation and its environmental impacts, waste cooking oil (WCO) has emerged as both a pollutant and a valuable renewable feedstock. WCO can be efficiently converted into sustainable surfactants with favorable surface and interfacial properties suitable for reservoir conditions. In this study, anionic and nonionic surfactants were synthesized from WCO: ethoxylated dodecylbenzene sulfonate (EABS14, aromatic) and ethoxylated hydrolyzed waste oil (EHWO14, aliphatic). Isopropanol was incorporated as a co-surfactant to enhance microemulsion performance. Phase behavior was evaluated at salinities of 50 × 10³, 100 × 10³, and 200 × 10³ ppm and at temperatures of 50 and 70 °C. Solubilization parameters and microemulsion phase volumes were used to determine optimal conditions. At optimum salinity, the dynamic interfacial tension (IFT) decreased significantly, reaching 1 × 10⁻³, 1 × 10⁻², and 8 × 10⁻² mN/m for EHWO14 + EABS14+CS, EHWO14, and EHWO14 + EABS14, respectively. Contact angles were reduced from 155° to 27°, 35°, and 22°, indicating effective wettability alteration. Flooding experiments confirmed the efficiency of WCO-derived surfactants, with maximum oil recovery at 100 × 10³ ppm and 50 °C. Recovery factors were 71.00% for EHWO14, 73.33% for EHWONa, 72.91% for EHWONa+EHWO14, and highest at 79.00% for the EHWO14 + EABS14+CS blend. These results demonstrate that WCO valorization into surfactants provides an eco-friendly and effective EOR alternative.

**Supplementary Information:**

The online version contains supplementary material available at 10.1038/s41598-026-55003-z.

## Introduction

The oil and gas sectors are looking for improved oil recovery strategies to increase the recovery of oil from reservoirs. Injecting chemical slugs into oil reservoirs is a well-established EOR method^1–4^. The most common variants of this method involve injecting slugs containing surfactant, alkaline surfactant, or alkaline-surfactant-polymer (ASP)^5–9^. By adjusting the injected fluid’s parameters, each additive in the chemical slug enhances the EOR mechanisms, such as interfacial tension (IFT) reduction, wettability alteration, and mobility ratio control^10–13^. Microemulsions play a key role in enhanced oil recovery (EOR) by significantly reducing the interfacial tension (IFT) between oil and water, enabling the mobilization of trapped oil within reservoir pores by overcoming capillary forces. During microemulsion flooding, surfactants and co-surfactants are injected to form a middle-phase system that can achieve ultra-low IFT values (~ 10⁻³–10⁻⁴ mN/m). Their effectiveness is attributed to small droplet size, large surface area, and favorable solubility and penetration properties. Microemulsions are commonly evaluated through core flooding, stability tests, and IFT measurements. According to Winsor classification, they are categorized into four types: type I (oil-in-water), type II (water-in-oil), type III (bicontinuous middle phase), and type IV (single-phase). Among these, types III and IV provide the most effective IFT reduction and are therefore more favorable for EOR applications^14,15^. Low interfacial tension (IFT) indicates high mutual solubility between oil and water phases. For effective EOR development, three critical factors must be addressed: accurate characterization of reservoir rock wettability, optimal control of surfactant adsorption, and efficient IFT reduction at oil-water interfaces. Surfactant chemistry plays a pivotal role in achieving these interfacial modifications by controlling molecular interactions at fluid-fluid and fluid-rock interfaces. Surfactant molecules are essential for controlling IFT in enhanced oil recovery applications. Surfactant molecules modify interfacial molecular interactions through adsorption at liquid-liquid interfaces, reducing repulsive forces and stabilizing the system by minimizing interfacial energy. The aqueous solution contains surfactant monomers that adsorb at interfaces, forming a monolayer which reduces both surface and interfacial tension. At specific concentrations, surfactant monomers form a closed aggregate (micelle), with the hydrophobic tails preserved from water and the hydrophilic heads facing it. This transition point is defined as the critical micelle concentration (CMC), a key surfactant property^16,17^. Surfactant injection has been successfully implemented in oilfield applications. Optimizing both concentration and selecting appropriate surfactants as key components of chemical slugs are critical for effectively mobilizing residual oil trapped in reservoir pore spaces. The surfactants are typically introduced into the injected water to achieve significant interfacial tension reduction between oil and water phases. Since surfactant performance and stability vary significantly with reservoir conditions, comprehensive EOR laboratory testing is essential to quantify surfactant efficiency prior to field implementation. The application of bio-based surfactants has gained significant attention in recent years. Researchers are increasingly investigating novel natural sources for surfactant production, motivated by their cost-effectiveness, abundant availability, and environmental sustainability^18–23^. For example, a castor oil-derived surfactant demonstrated exceptional performance by achieving ultra-low interfacial tension and effectively altering sandstone wettability from oil-wet to strongly water-we^3,23,24^. The surfactants have stable foam and emulsion properties^25^. To overcome the drawbacks of chemical surfactants, such as their high cost and environmental incompatibility, research into bio-based surfactants is ongoing. From both scientific and commercial perspectives, biosurfactants offer benefits such as sustainability and environmental friendliness^26^. Additional benefits of natural surfactants include low toxicity, high biodegradability, and greater accuracy and efficacy at high pH and temperature^27^. Various studies on the use of waste materials in EOR were reviewed, including those on environmentally safe potassium-doped graphene oxide particles made from oak fruit waste. These raw materials are abundant, cost-effective, biocompatible, biodegradable, and readily accessible. Interfacial tension, critical micelle concentration, and zeta potential were measured to assess the stability of particles treated with different surfactants. Adsorption experiments subsequently quantified surfactant loss during the flooding process^28^. A further investigation found that reduced graphene oxide, synthesized using waste plastic as a carbon source, enhanced the EOR process. These materials exhibit superior electrical, mechanical, and thermal properties because the synthesized graphene has a larger surface area than conventional metal oxide nanoparticles, resulting in higher surface activity. Converting solid waste into valuable carbon-based nanomaterials is a vital strategy for solid waste management^29^. Additionally, red onion skin, an agricultural waste biomass, was chosen as a natural recovery agent. This agricultural waste is a crucial source of quercetin, flavones, anthocyanins, and polyphenolics. Red onion skin was treated with glutaraldehyde in an aqueous sodium hydroxide solution and its efficacy was assessed in a sandstone core^30^. Another study was conducted to investigate the interactions of a biopolymer (animal glue) and a surfactant (lauryl ether sulfate) with hydrolyzed polyacrylamide. Several laboratory floods using steam, surfactant, biopolymer, and polymer were conducted individually and in combination on a semi-pilot model^31^. Many studies offer a critical evaluation of various green innovations for enhanced oil recovery, including organic materials injection, nanotechnology application, solar energy integration, microbial processes, water alternating gas, foam injection, and nanofluid injection, all contributing to a cleaner environment^32^. Researchers focus on the various advantages of green nanocomposites in EOR, primarily their safety for living things. As they are derived from plants or other safe industrial resources, these nanocomposites represent a highly attractive alternative EOR technique^33^. In another study, zinc oxide nanoparticles, which can prevent asphaltene deposition, reduce interfacial tension, and alter wettability, were combined with green nanocomposites in porous media for enhanced oil recovery^34^. Waste cooking oil (WCO) is a non-edible oil produced in large amounts. Its careless disposal may have a detrimental effect on humanity and the environment^35^. To improve the clarity and scientific rigor of this study, the objectives are formulated as specific, testable research questions. This work aims to investigate how the molecular characteristics of the synthesized bio-based surfactant, derived from waste cooking oil as a renewable resource, influence its interfacial properties, particularly in terms of critical micelle concentration (CMC) and water–oil interfacial tension (IFT) reduction. Furthermore, the study examines the effect of salinity and formulation conditions on microemulsion phase behavior and the determination of optimum (ideal) salinity. In addition, the relationship between wettability alteration, interfacial tension reduction, and their combined impact on enhanced oil recovery (EOR) efficiency is systematically evaluated using a sand-pack model. This research provides a clear framework to establish the link between surfactant structure, physicochemical performance, and oil recovery efficiency. Recent studies on bio-based and waste-derived surfactants for EOR have shown promising results; however, many systems still require alkali addition, high concentrations, or complex formulations to achieve ultra-low interfacial tension. In this context, the present work introduces a synergistic WCO-derived surfactant system combining aromatic (EABS14) and aliphatic (EHWO14) components with a co-surfactant, enabling ultra-low IFT (down to 10⁻³ mN/m) without alkali under moderate conditions. The system also demonstrates high oil recovery efficiency (up to 79%), highlighting its potential as a more effective and practical alternative to existing bio-based surfactant systems.

## Materials and methods

### Materials

#### Surfactants

The surfactants used in this study were prepared from waste cooking oil. The anionic and nonionic surfactants used in this study were synthesized from waste cooking oil (WCO)-derived feedstock following the procedure reported in our previous work. In brief, the synthesis involved controlled chemical modification of the WCO-derived precursor under optimized reaction conditions to obtain the target surfactant structures. After completion of the reaction, the products were purified using standard separation and washing procedures to remove unreacted species and residual impurities. The obtained surfactants were then dried and stored for further use. The chemical structure and successful functionalization of the synthesized surfactants were confirmed in the previous study which verified the formation of the desired amphiphilic structures. Therefore, only a summary of the synthesis route is presented here, while full experimental details and characterization data are available in the cited reference^36^. The surfactants used in this study are: Ethoxylated Hydrolyzed Waste Oil (eo = 9) [EHWO9], Ethoxylated Hydrolyzed Waste Oil (eo = 14) [EHWO14], Ethoxylated Hydrolyzed Waste Oil (eo = 23) [EHWO23], Ethoxylated Hydrolyzed Waste Oil (eo = 46) [EHWO46], and Hydrolyzed Waste Oil sodium salt [HWONa].

#### Crude oil

The crude oil used for preparing microemulsions was sourced from the West Desert, Egypt, and provided by the General Petroleum Company (GPC). The oil had an API gravity of 27° at 15.55 °C and a density of 0.8983 at 15 °C. Its composition, determined by SARA analysis, was 57 wt% saturates, 21 wt% aromatics, 15 wt% resins, and 7.63 wt% asphaltenes.

#### Formation water

Formation water, obtained from GPC, was used to prepare microemulsions under reservoir conditions. The water had a total dissolved solids (TDS) content of 200 × 10³ ppm. This formation water was diluted with distilled water to achieve lower salinities of 50 × 10³ and 100 × 10³ ppm.

#### The used Sand in the Sand-Packed Model

Sand of various sizes (12, 18, and 20 mesh, corresponding to 1.68, 1.00, and 0.841 mm, respectively) was used to pack the model for the chemical flooding test to achieve a porosity of 25%.

### Methods

#### The used surfactant

Previous work involved preparing and characterizing surfactants extracted from waste cooking oil. The investigation included measuring surface tension, interfacial tension (IFT), estimating the critical micelle concentration (CMC), and determining thermodynamic properties^36^.

#### Preparation of microemulsions

The procedure for microemulsion stability testing was as follows. Surfactant solutions were prepared at a concentration of 0.25% in formation water with salinities of 50 × 10³ ppm, 100 × 10³ ppm, and 200 × 10³ ppm. Equal volumes of crude oil and surfactant solution were placed in graduated test tubes with rubber caps. The tubes were shaken by hand 60 times at a steady rate (completed within one minute) and were then allowed to settle for 24 h. To investigate the impact of temperature, duplicate sets of tubes for each salinity were incubated at 25 °C, 50 °C, and 70 °C. The entire experiment was performed in triplicate to ensure reproducibility. All microemulsion stability tests were initially performed in triplicate to ensure reproducibility. In cases where phase behavior variations were observed, additional repetitions were carried out until consistent and stable microemulsion behavior was confirmed under each experimental condition. The results reported in this study therefore represent reproducible and stable phase observations rather than single-run measurements. Since the evaluation is based on qualitative and semi-quantitative phase behavior (i.e., microemulsion formation, stability, and phase separation).

#### Solubilization parameters and relative phase volume

The phase behavior of the microemulsions was investigated using solubilization parameters. These parameters were calculated based on the volume of oil and water solubilized in the middle phase under ambient conditions. In the test tubes, a three-phase system was observed: an aqueous phase at the bottom, a microemulsion phase in the middle, and an oil phase at the top. This middle microemulsion phase is effective for oil recovery due to its ultra-low interfacial tension (IFT), which enables it to solubilize trapped oil from reservoir porous media. The solubilized oil volume was calculated using Eq. 1.

1 $$Vo\,=\,{V_{oi}} - {V_{of}}$$

Where Vo represents the volume of solubilized oil in the microemulsion region, V_oi_ represents the initial volume of the used oil, and V_of_ represents the volume of oil in the upper phase. Similarly, the volume of water can be calculated using Eq. 2.

2 $$Vw\,=\,{V_{wi}} - {V_{wf}}$$

Where Vw denotes the volume of solubilized water in the microemulsion region, Vwi denotes the initial volume of the used water, and Vwf denotes the volume of water in the lower phase. The solubilization values of oil and water were calculated using Eqs. 3, 4.

3 $$SPo\,=\,Vo/Vs$$

4 $$SPw\,=\,Vw/Vs$$

Where SPo and SPw are the solubilization parameters of oil and water, and Vs is the volume of surfactant in the middle phase. The surfactant volume, Vs, is constant in the middle-phase microemulsion. On a solubilization curve, the point where the oil and water solubilization ratios are equal is crucial, as it identifies the optimal salinity. At this optimal salinity, equal amounts of oil and water are solubilized into the middle phase, forming a Winsor type III microemulsion system.

#### Phase diagram

The solubilization method was used to construct triangular pseudo-ternary phase diagrams at room temperature (25 °C). Surfactant (at a fixed mass), oil, and brine (pseudo-components) made up the triangle’s three vertices. The experiments were set up using volumetric measurements, and the results were expressed as weight percentages.

#### Oil-water IFT

Interfacial tension (IFT) between crude oil and surfactant solutions was measured at critical micelle concentration (CMC) and 50 °C. To determine the optimal salinity, IFT tests were conducted at various salinity concentrations (50 × 10^3^ ppm, 100 × 10^3^ ppm, and 200 × 10^3^ ppm).

#### Contact angle experiments

The wettability of the reservoir model was examined by measuring the contact angle between crude oil and sand particles. These measurements, which indicate wettability alteration, were conducted using a High-Pressure Chamber (Attention Theta model) via the sessile drop method (ASTM/ISO 19403-5). The device measured the contact angle at the interfaces between the crude oil, brine/surfactant solution, and the core surface. All measurements were conducted at a constant temperature of 50 °C. Sandstone samples were cut into thin sections approximately 2.5 mm thick to observe the penetration and spreading of oil droplets. The sections were first cleaned with high-pressure nitrogen gas and then aged in toluene solvent for 24 h to remove contaminants, such as fatty acids from hand contact. Finally, to achieve a hydrophobic surface, the sections were heated to 50 °C in crude oil. Contact angle measurements were performed at the critical micelle concentration (CMC) of the surfactant solution and recorded at various time intervals. A section holder was used to suspend the rock sample in the aqueous medium, positioning it a short distance from the needle. The associated software then automatically calculated the contact angle by drawing tangent lines to the droplet at the rock surface interface.

#### Emulsion stability

The stability of emulsions prepared with the surfactant solution was tested at its critical micelle concentration (CMC), with varying salinity and co-surfactant concentrations, and at different temperatures. Test tubes containing the prepared microemulsions were placed in water baths at 50 °C and 70 °C to monitor their stability over time.

#### Surfactants slug injection

**Fig. 1:**
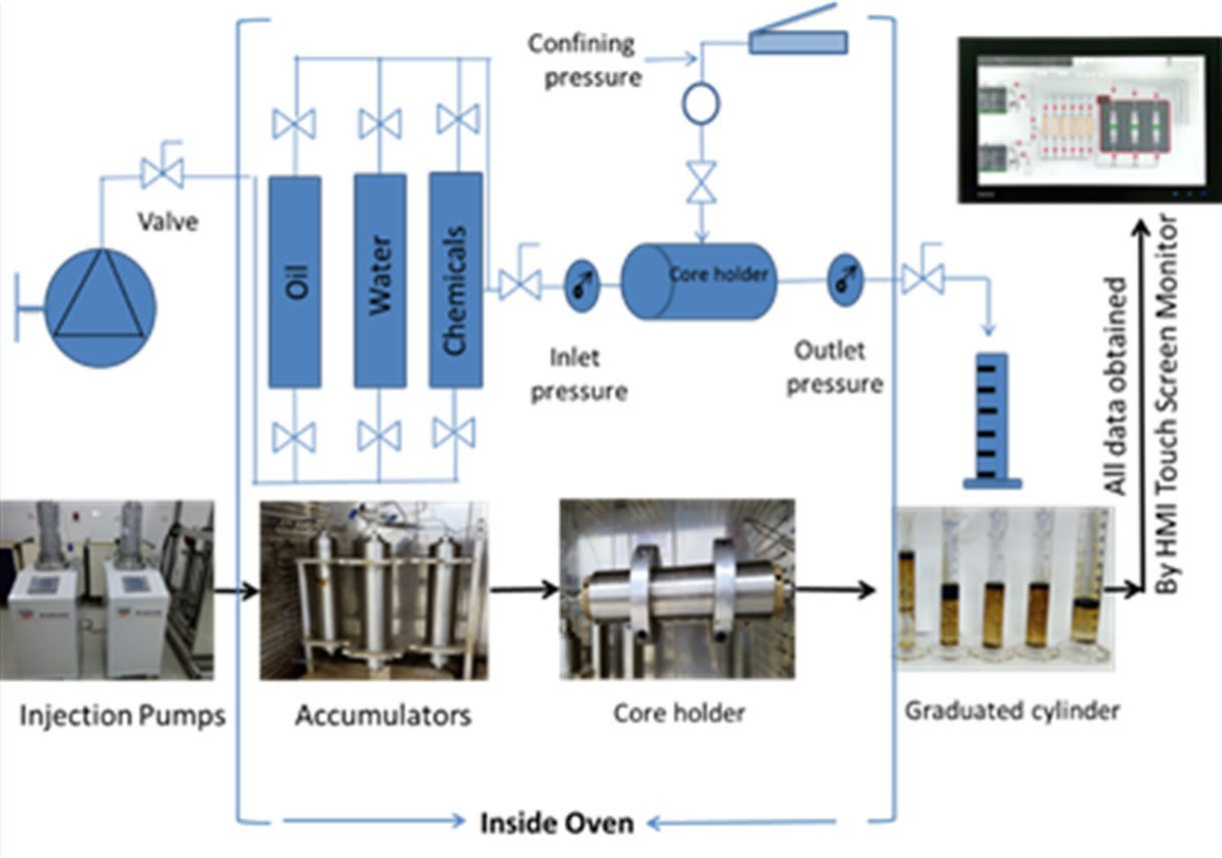
Schematic Setup of Chemical Flooding Process.

The core flooding procedure was conducted as follows: the model was first saturated with water. This was followed by oil injection to establish initial oil saturation, and then brine flooding until a 98% water cut was achieved. Next, a 1 PV surfactant slug—prepared at the critical micelle concentration (CMC) and the reservoir’s optimum salinity—was injected. Finally, brine was re-injected until the water cut reached 99%. A chemical flooding system was used to evaluate the performance of the green microemulsion. A schematic diagram of the surfactant flooding apparatus is shown in Fig. 1. The system’s main components include three cylinders, an injection pump, a core holder, a back-pressure regulator, and a produced fluid collection container. The cylinders and the core holder were housed inside an oven to maintain the system temperature at 50–70 °C. During the test, the injection rate was set to 2 cm³/min. To inject a specific fluid, the valves for the other cylinders were closed, and the valve for the desired fluid was opened.

#### Water flooding (secondary recovery)

During water flooding, water was injected at a constant rate of 3 cm³/min until oil production from the sand pack model became negligible (less than 1 cm³). The produced oil and water were collected in graduated tubes, and their volumes were recorded. Furthermore, water breakthrough times were observed. At the end of the water flooding process, the residual oil saturation was determined.

#### Surfactant flooding (tertiary recovery)

The sand pack model was heated to the reservoir temperature of 50–70 °C. Upon reaching the desired temperature, the surfactant solution was injected at a rate of 3 cm³/min, followed by saline water injection until oil production ceased. The tests were conducted at an optimum surfactant concentration of 0.25% and salinities of 50 × 103, 100 × 103, and 200 × 103 ppm. For each surfactant slug size, the produced oil and water were collected in graduated cylinders and their volumes were recorded. Water breakthrough times were also measured for the different slug sizes. The properties of the reservoir model are provided in Table 1.

**Table 1:** Properties of Reservoir Model.

Property	Value
Reservoir type Diameter (mm) Length (mm) Surface (cm²) Bulk volume (cc) PV known (cc) ρ fluid (g/cc) Porosity (%)	Sandstone 50.00 300.00 19.63 589.28 150.00 1.00 25.00

#### Reservoir relevance and limitations

Although the sand-packed model provides a controlled and reproducible medium for evaluating surfactant performance, it represents a simplified approximation of actual reservoir systems. Sand packs primarily mimic homogeneous, silica-based porous media and therefore do not fully capture the mineralogical complexity, surface heterogeneity, and pore structure variability present in real sandstone and carbonate reservoirs. In particular, carbonate formations with positively charged surfaces and clay-rich sandstones may exhibit stronger surfactant adsorption, which can reduce the effective concentration of the injected chemicals and influence overall efficiency.

Furthermore, adsorption losses in real reservoir rocks are expected to be higher than those observed in sand-packed systems, potentially altering optimum salinity conditions and microemulsion behavior. Permeability heterogeneity in reservoir formations may also impact fluid flow, leading to channeling or early breakthrough, which is not adequately represented in the uniform sand-pack model.

Despite these limitations, the sand-packed flooding experiments provide valuable insight into the intrinsic displacement efficiency of the developed surfactant systems, particularly in terms of interfacial tension reduction and wettability alteration. However, for practical field application, further validation through core flooding experiments using representative reservoir rocks under actual reservoir conditions (temperature, pressure, and salinity) is required. This step is essential to assess adsorption behavior, injectivity, and large-scale displacement efficiency.

## Results and discussion

### Investigation of phase behavior and solubilization parameters

**Fig. 2:**
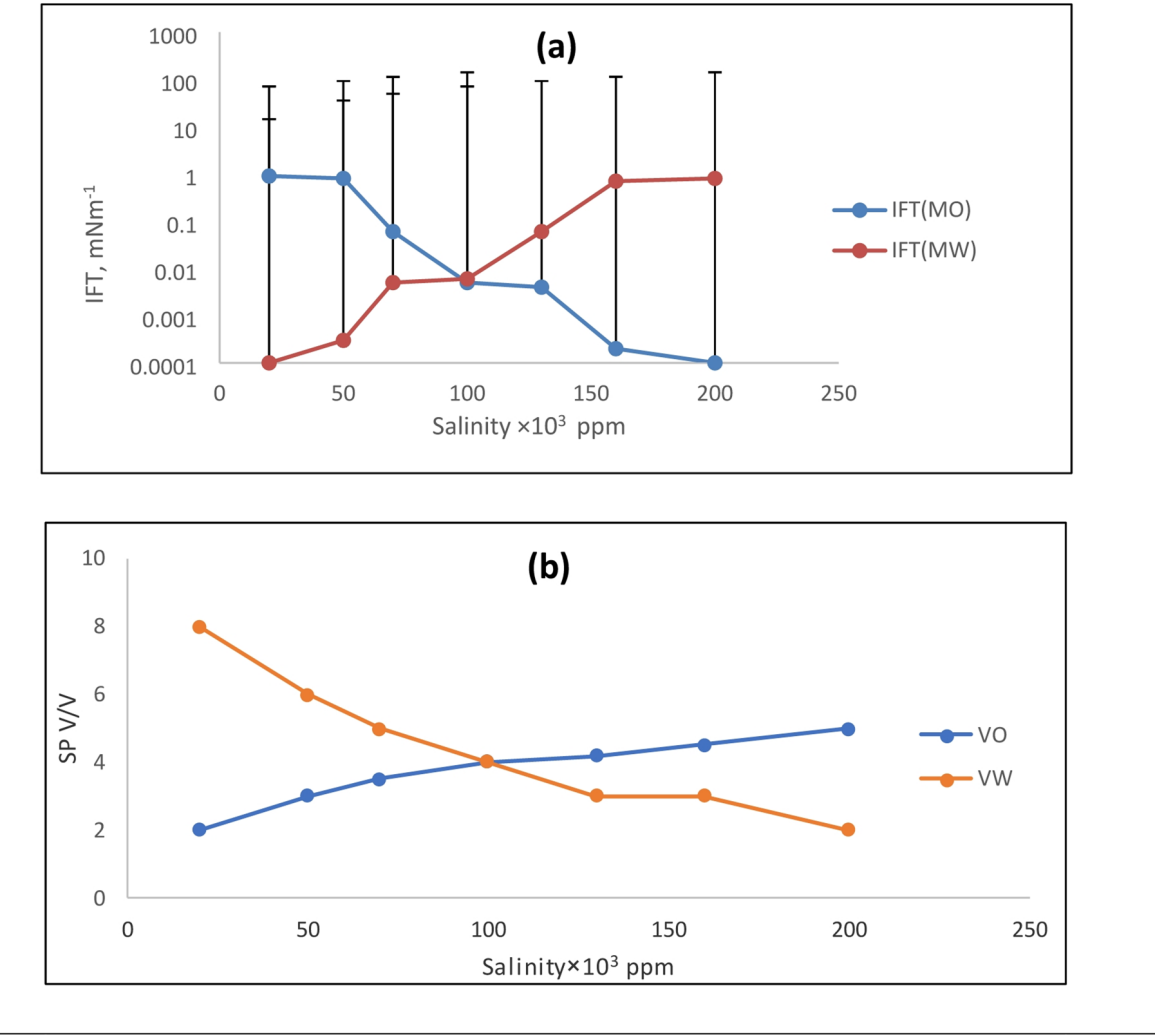
(**a**) Interfacial Tension vs. Salinity; (**b**) Solubilization Parameters vs. Salinity for EHWO14.

**Fig. 3:**
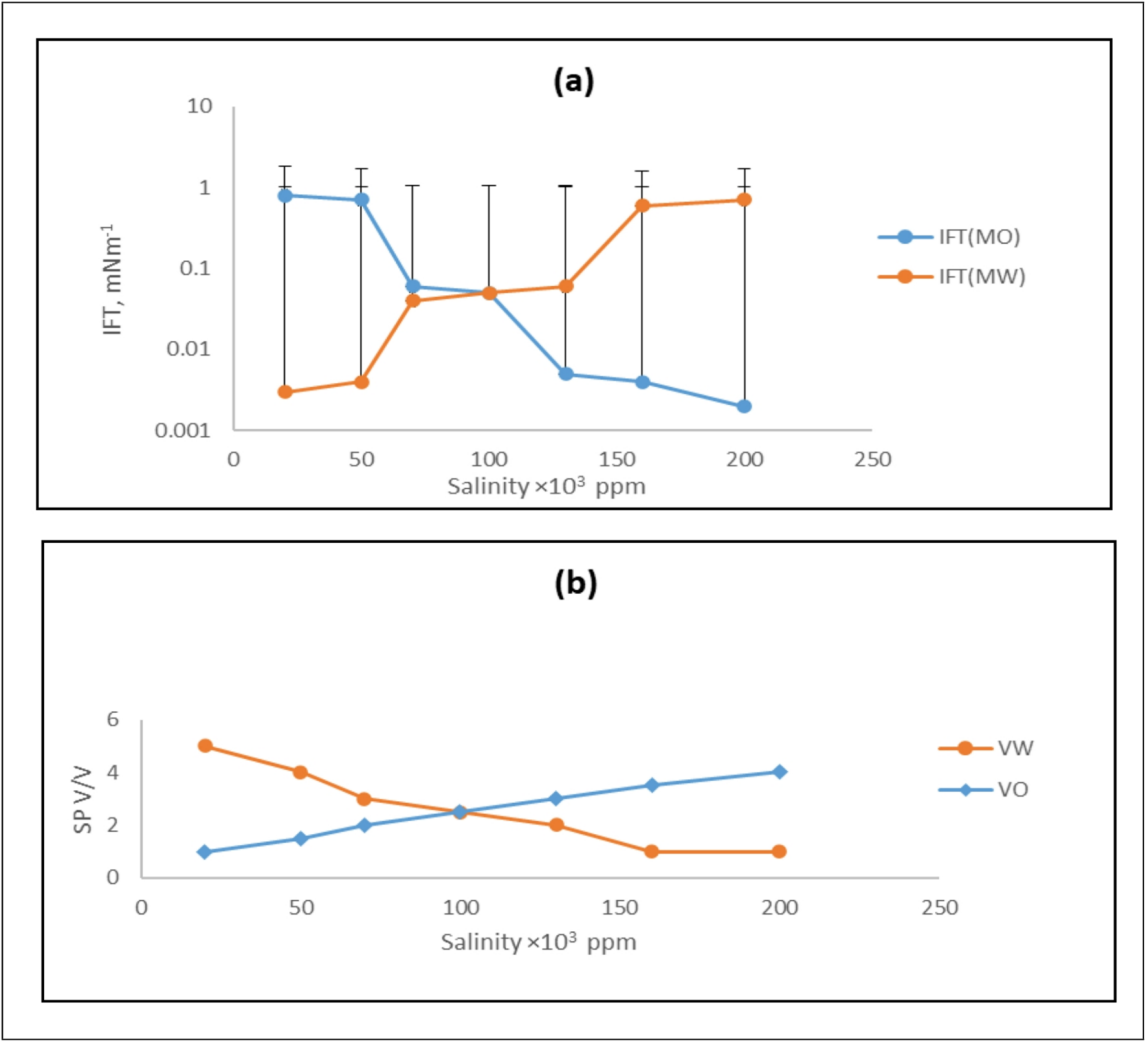
(**a**) Interfacial Tension vs. Salinity; (**b**) Solubilization Parameters vs. Salinity for EHWO14 + EABS14+Cs.

**Fig. 4:**
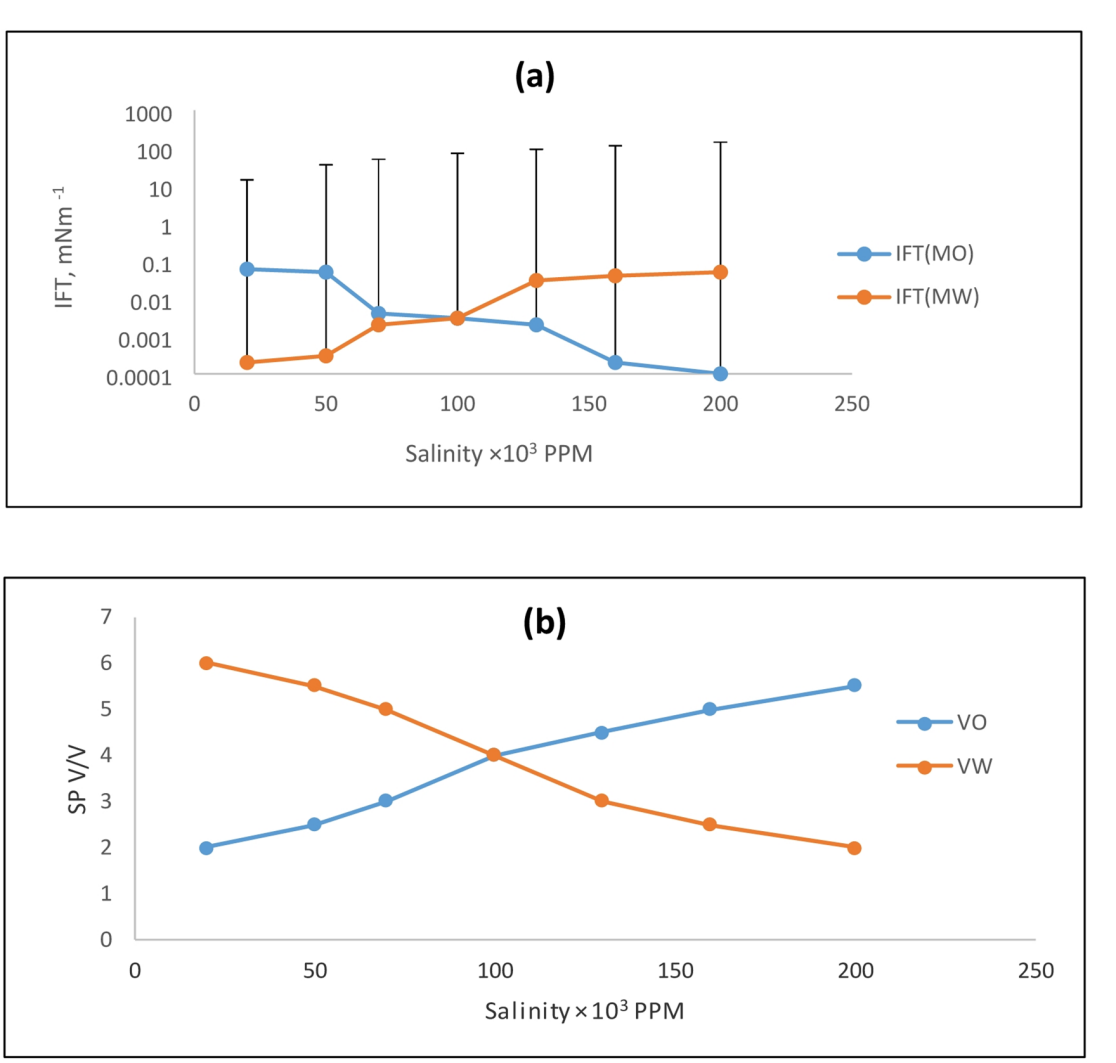
(**a**) Interfacial Tension vs. Salinity; (**b**) Solubilization Parameters vs. Salinity for EHWONa.

The most crucial factor in determining the success of chemical flooding is the phase behavior of the mixture of brine, crude oil, and surfactant. In these surfactant systems, achieving ultralow interfacial tension (IFT) is critical, as it is effective for studying the phase behavior of microemulsions, particularly in regions of high solubilization. The phase behavior of the system is primarily governed by surfactant concentration, crude oil properties, and brine salinity. Additionally, the effect of high temperature under typical reservoir conditions is a critical parameter. Therefore, the main aim of this study is to determine the combined effects of salinity and temperature on the phase behavior of the surfactant-brine-crude oil system for enhanced oil recovery (EOR) applications. The phase behavior was investigated to determine the effect of increasing salinity and temperature on the stability of the surfactant-brine-crude oil emulsion. The volumes of solubilized water and oil in the microemulsion system were used to determine the solubilization parameters for the Winsor type III microemulsion. The oil solubilization parameter (Vo/Vs) increased with salinity, while the water solubilization parameter (Vw/Vs) decreased. At low salinity, the majority of the surfactant and co-surfactant resided in the aqueous phase, with only a trace amount solubilized in the oil phase. At high salinity, only a small amount of surfactant was soluble in the aqueous phase, with the majority residing at the interface. As salinity increased, the oil solubilization parameter increased, while the water solubilization parameter decreased. The salinity at which these two values became equal is known as the optimal salinity. The optimal salinity depends on the crude oil type, the surfactant-to-co-surfactant ratio, and temperature. For the EHWO14 system, Fig. 2 shows the interfacial tension (IFT) and solubilization parameters as functions of salinity. Figure 3 depicts the same relationships for a blend of EHWO14 with EABS14 and isopropanol as a co-surfactant (denoted as EHWO14 + EABS14+Cs). Furthermore, Fig. 4 shows the interfacial tension (IFT) and solubilization parameters as functions of salinity for the EHWONa system. The experimental results indicate that salinity increases solubilization parameters up to a maximum value, beyond which they decrease. In this study, the optimal salinity was determined to be 100 × 10³ ppm. At this optimum salinity, the middle-phase microemulsion solubilizes equal volumes of oil and water. After exceeding the optimal salinity, a further increase in salt concentration to 200 × 10³ ppm decreased the water solubilization parameter. Concurrently, the microemulsion system transitioned from Winsor type I to Winsor type III, and finally to Winsor type II as salinity increased. These phase transitions can be explained by the interaction of inner droplets and interfacial bending stress. Specifically, increasing salt concentration attracts water molecules to solvate the ions. This reduces the number of water molecules available to hydrate the hydrophilic head groups of the surfactant, increasing the attraction between the head groups and reducing the effective cross-sectional area of the surfactant’s hydrophilic head. Consequently, the natural curvature of the interface changes, promoting a transition toward a water-in-oil (Winsor type II) microemulsion. The curvature of the interfacial film transitions from positive, to zero, to negative, corresponding to the microemulsion phase transition from oil-in-water (O/W, Winsor type I), to bi-continuous (Winsor type III), to water-in-oil (W/O, Winsor type II). Consequently, with increasing salinity, the microemulsion system undergoes a transition where the surfactant-rich phase shifts from the lower (O/W) to the middle (bi-continuous) to the upper (W/O) phase. Accordingly, the oil solubilization parameter (Vo/Vs) increases with salinity, while the water solubilization parameter (Vw/Vs) decreases^37–42^.

### Phase diagram of the micro emulsion system

**Fig. 5:**
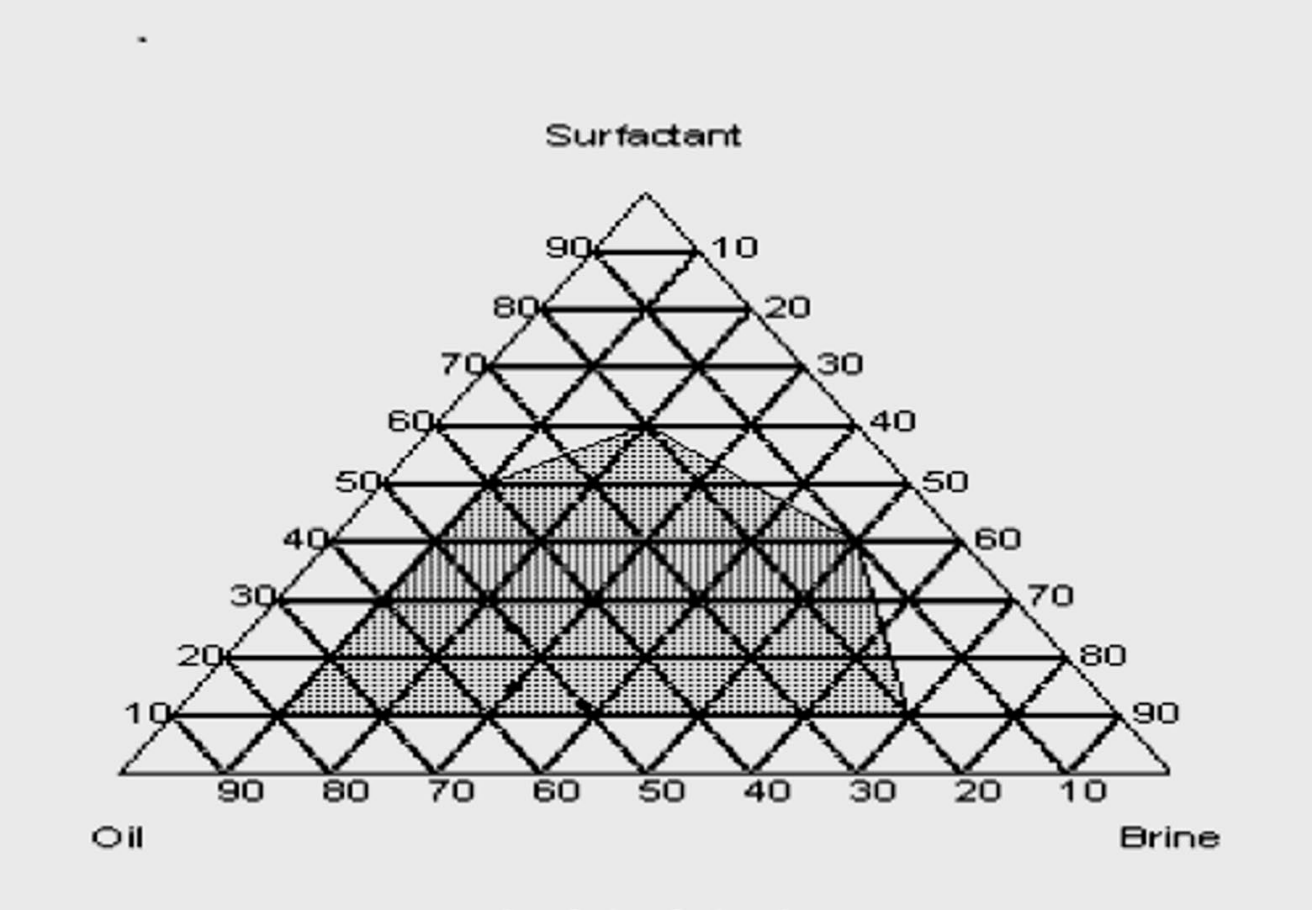
Ternary Phase Diagram of Oil-Brine- Surfactant (EHWO14) System.

**Fig. 6:**
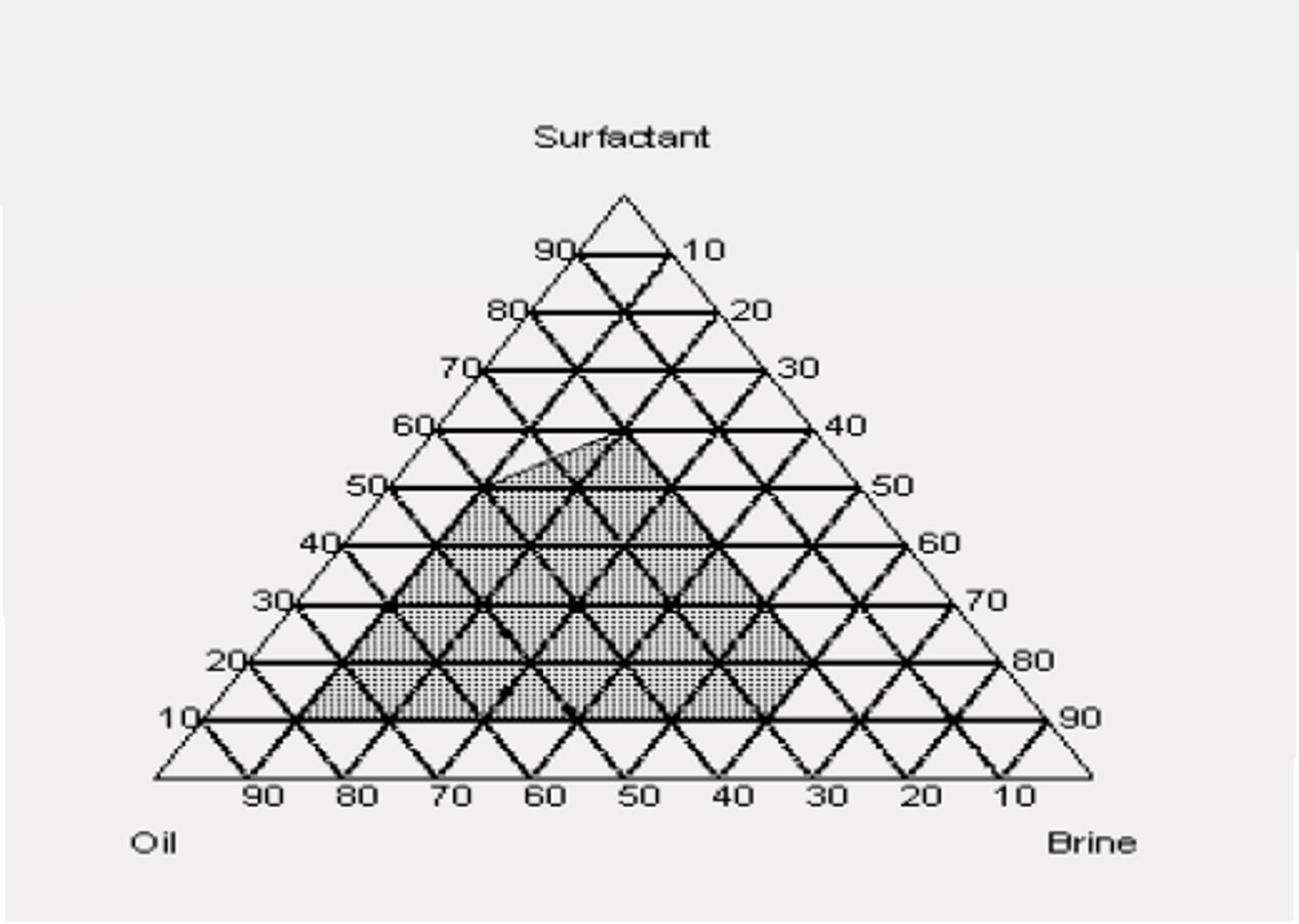
Ternary Phase Diagram of Oil-Brine- Surfactant Blend (EHWO14 + EABS14+Cs) System.

**Fig. 7:**
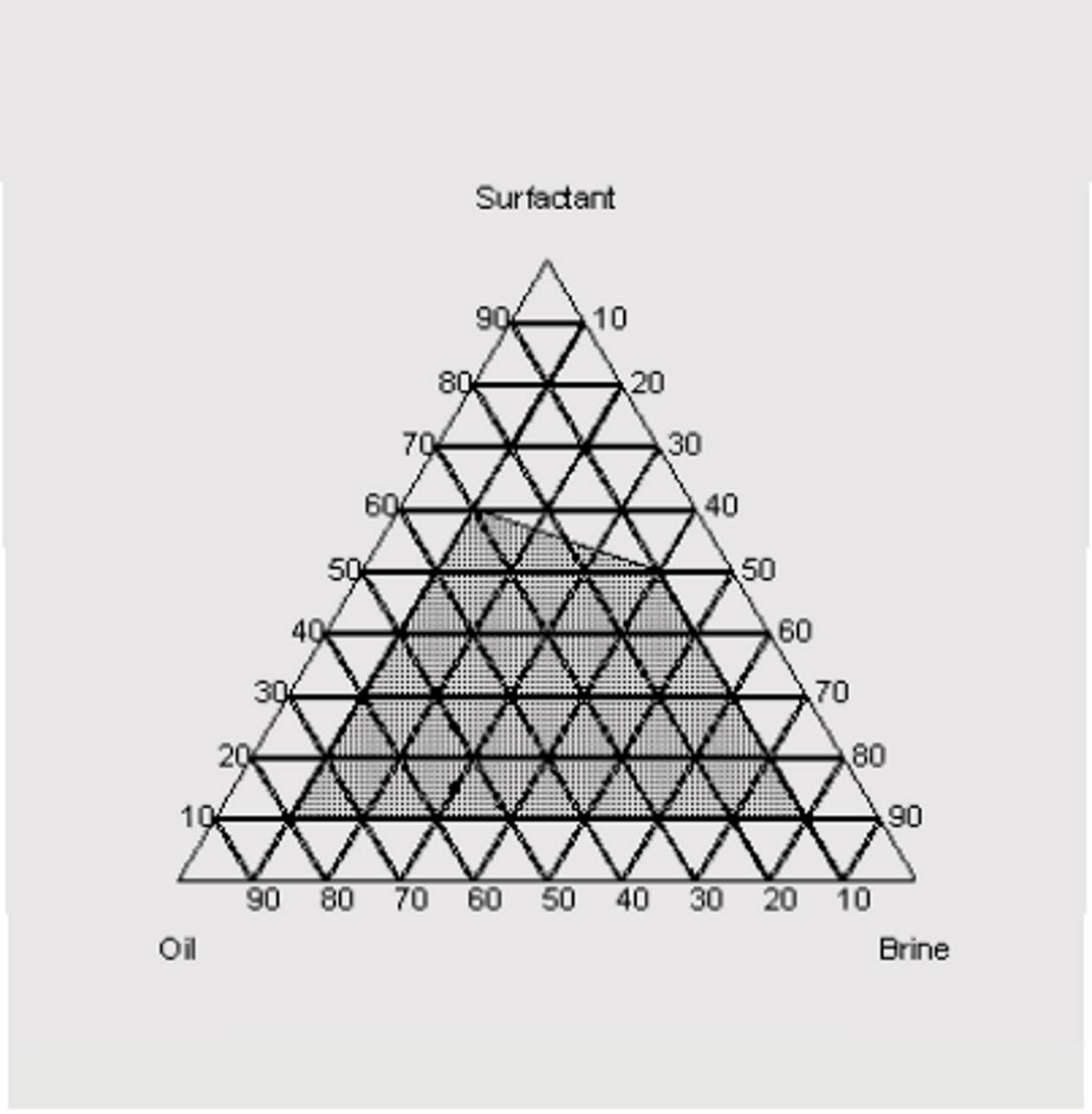
Ternary Phase Diagram of Oil-Brine- Surfactant (EHWONa) System.

**Fig. 8:**
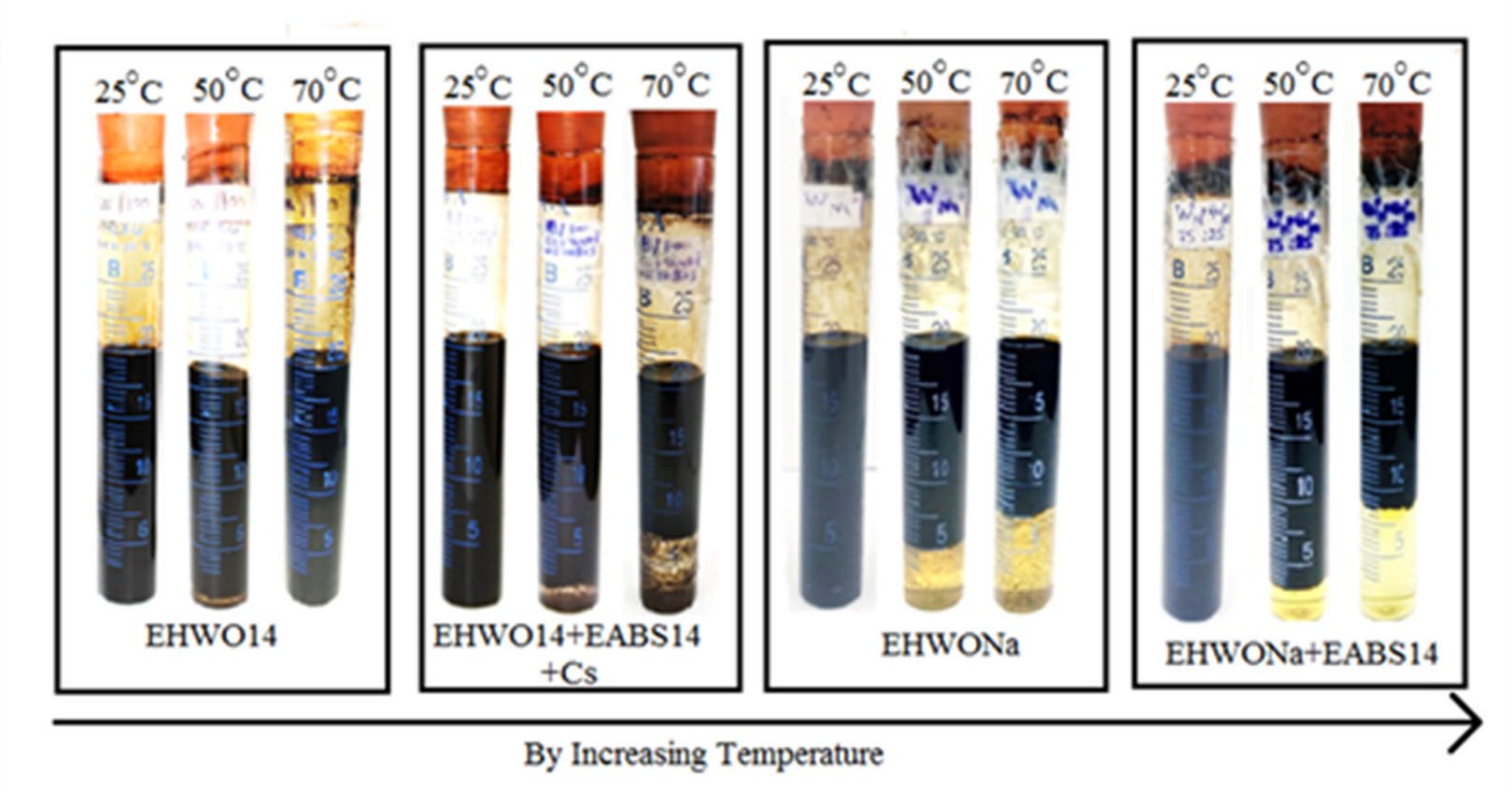
Phase Behavior of EHWO14 and Its Derivatives at Optimal Salinity and Different Temperatures.

Pseudo-ternary diagrams were constructed with brine, crude oil, and a surfactant/co-surfactant mixture (S + Cs) as the three components. These diagrams were established for various surfactants to determine the composition of the microemulsion phase. Furthermore, formulating microemulsions with a low surfactant concentration is essential for cost reduction. Figure 5 presents the corresponding pseudo-ternary diagram for the surfactant EHWO14, formation water, and crude oil, with the microemulsion region indicated by the shaded area. The pseudo-ternary diagram in Fig. 6, which features the surfactant blend (EHWO14 + EABS14+Cs), formation water, and crude oil, shows a larger microemulsion region than those of the individual surfactants (Figs. 5 and 6). Isopropanol, used as a co-surfactant, increases the solubility of surfactant molecules in the oil phase and enhances microemulsion stability. As shown in Fig. 7, the microemulsion region was more stable for the anionic surfactant (EHWONa) than for the individual nonionic surfactant or their blends. Figure 8 shows the phase behavior of EHWO14, its derivatives (EHWO14 + EABS14+Cs), and EHWONa at optimal salinity and different temperatures.

### Microscopic investigation of the micro emulsion phase volume

The experiments were carried out using EHWO14 at 25 °C in formation water with total dissolved solids (TDS) concentrations of 50 × 10³, 100 × 10³, and 200 × 10³ ppm. A middle-phase microemulsion formed at a 1:1 oil-to-water ratio, as confirmed by optical microscopy. However, this microemulsion gradually separated at salinities of 50 × 10³ and 200 × 10³ ppm when the temperature was increased from 25 °C to 70 °C. The microemulsion remained stable at a salinity of 100 × 10³ ppm across all three temperatures. As the temperature increased, the oil viscosity decreased, leading to the agglomeration of oil droplets in the upper phase. The blend (EHWO14 + EABS14+Cs) was used to prepare the microemulsions at 25 °C and three different salinities. After settling, the largest microemulsion region was observed at the salinity of 100 × 10³ ppm. A microemulsion formed at salinities of 50 × 10³ and 100 × 10³ ppm, but the region decreased at 200 × 10³ ppm. This behavior is likely due to achieving a minimum interfacial tension of 2 × 10⁻² mN/m. As the temperature increased, the microemulsion region gradually decreased. However, analysis of microscopic data for the anionic surfactant EHWONa at the optimal salinity of 100 × 10³ ppm showed that the microemulsion region was largest and most stable at 50 °C. Microemulsions are highly sensitive to elevated temperatures; this thermal instability must be balanced during the formulation of a surfactant flooding process^43–48^.

### Factors effect on phase behavior

#### Molecular structure

Molecular structure is one of the most crucial factors affecting surfactant performance. In this study, modifications to the molecular structure played a vital role in phase behavior. The data show that anionic surfactants are more efficient than nonionic surfactants when used individually, while surfactant blends produced the most stable microemulsions.

#### Surfactant concentration

Surfactant concentration was maintained at the critical micelle concentration (CMC), as this value represents the point of maximum efficiency. However, for the flooding process, the surfactant was injected at a concentration slightly above the CMC to compensate for losses due to adsorption onto the rock surface.

#### Cosolvents

Co-solvents, like isopropanol, increase microemulsion stability by enhancing the solubility of surfactant molecules in the oil phase.

#### Salinity

Salinity significantly influenced the phase behavior, with an optimal salinity observed at 100 × 10³ ppm. Increasing the salinity raised the oil solubilization ratio while decreasing the water solubilization ratio. In distilled water, a balance exists between monolayer surfactant adsorption at the interface and micellization. Furthermore, hydrogen bond formation induces significant coiling of the ethylene oxide groups, which increases the minimum area per molecule (A_min_) and decreases the maximum surface concentration (Γ_max_). Conversely, in formation water, the high salt concentration disrupts hydrogen bonding, which reduces A_min_ and increases Γ_max_. This mechanism is critical for enhanced oil recovery (EOR), as the increased oil solubilization at the interface improves the overall efficiency of the process.

#### Temperature

Temperature is a critical parameter for surfactant-based enhanced oil recovery due to its significant influence on surfactant molecules at the interface and within the bulk brine phase. Figure 8 shows the effect of temperature on microemulsion phase behavior. As temperature increases, thermal agitation reduces the number of surfactant molecules at the interface, thereby increasing the minimum area per molecule (A_min_). However, at specific temperatures, the formation of thermodynamically stable microemulsions can improve oil displacement and increase the EOR efficiency. While the volume fraction of the aqueous phase remains largely constant with increasing temperature, the volume of the middle phase decreases considerably as excess oil is released. This reduction continues up to a specific temperature, beyond which the middle-phase volume stabilizes^36^. There are also factors such as crude oil type and water-oil ratio.

### Contact angle and wettability alteration

**Fig. 9:**
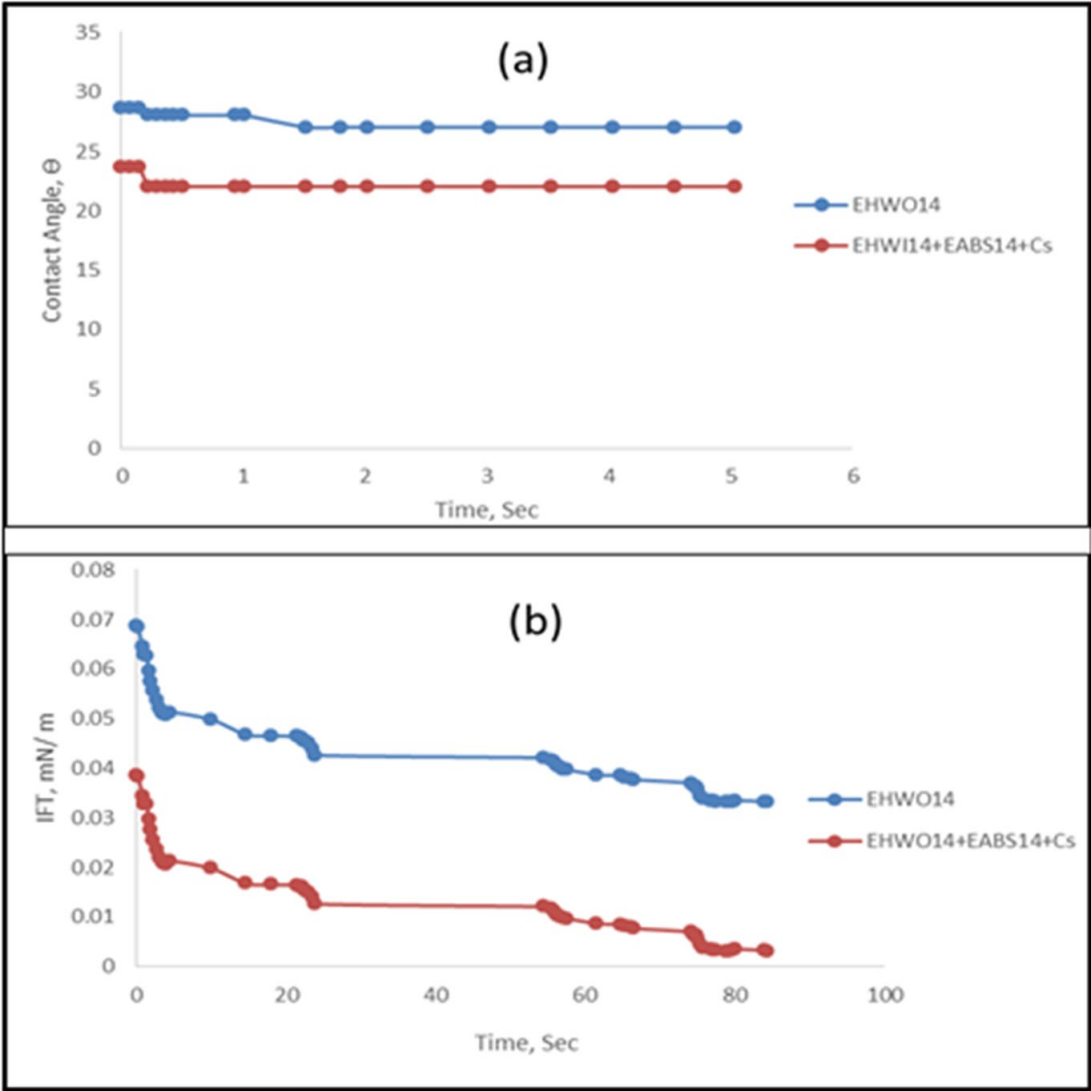
(**a**) Contact Angles vs. Time; (**b**) IFT vs. Time for EHWO14 and. its Blend (EHWO14 + EABS14+Cs) at 50 ^◦^C.

The contact angle of crude oil is influenced by surface charge density. Figure 9 illustrates the contact angle for crude oil on clean, loose sandstone samples (Arenite with mineralized SiO₂) treated with the surfactant EHWO14 and its blend formulations (EHWO14 + EABS14+Cs). According to Table 2, the contact angle of the untreated oil droplet was highly obtuse (159°), indicating an oil-wet rock phase. Treatment with surfactant formulations shifted the wettability to a water-wet state. Surfactants facilitate this change by reducing the interfacial tension (IFT) and contact angle, thereby lowering the adhesion work required to separate oil droplets from the rock surface and mobilize them. This reduction in adhesion work is essential for enhancing oil recovery. The temporal evolution of the contact angle is termed the dynamic contact angle. The dynamic contact angle measurements as a function of time are presented in Fig. 9, where the time axis is expressed in seconds (s). The results show a rapid decrease in the contact angle during the initial stage, followed by a gradual approach to a stable value. This behavior indicates progressive wettability alteration until equilibrium is reached. It was observed that the contact angle attained a nearly constant value after approximately [insert time, e.g., 180 s], suggesting that equilibrium conditions were effectively established within the experimental timeframe. This dynamic behavior, demonstrating that the presence of surfactant in the flooding formulations consistently reduces the contact angle over time. When surfactant adsorbs onto the oil droplet, its stability decreases, promoting rupture. In this study, surfactant adsorption at the oil-water interface formed a monolayer that rendered the oil droplet surface more hydrophilic. This promotes the formation of low-viscosity oil-in-water emulsions, thereby improving oil displacement and enhancing EOR efficiency^49,50^.

**Table 2:** Physical Terms of EHWO14 and its Blends.

Surfactant	Designation	HLB	Static IFT (mN/m)	Dynamic IFT (mN/m)	Static Contact Angle(Ө)	Dynamic Contact Angle(Ө)
1- Ethoxylated Hydrolyzed Waste Cooking Oil (eo =14)	EHWO14	14.6	4 × 10^-2^	1 × 10^-2^	31	27
2- Ethoxylated Hydrolyzed Waste Cooking Oil (eo =14) + alkyl Benzene sulfonate (eo =14)	EHWO14 +EABS14	14.6	1 × 10^-1^	8 × 10^-2^	40	35
3- Ethoxylated Hydrolyzed Waste Cooking Oil (eo =14) + Ethoxylated alkyl Benzene sulfonate (eo =14) + Co Surfactants	EHWO14 +EABS14+Cs	12.4	1 × 10^-2^	1 × 10^-3^	25	22

### Work adhesion

Wettability is an essential property in many surfactant applications. The contact angle (θ) of a liquid on a solid surface is influenced by the reduction in surface tension induced by a surfactant. This angle is governed by the liquid’s surface tension, the solid’s surface energy, and the solid-liquid interfacial tension. Furthermore, the arrangement and alignment of surfactant molecules at the solid-liquid interface directly affect the work of adhesion (Wa). The extent of wetting can be described by the difference between the work of adhesion (Wa) and the work of cohesion (Wc). Both the work of adhesion and the contact angle are dependent on the composition and surface tension properties of the solid and liquid. The residual oil saturation decreases as the contact angle at the fluid/rock/oil interface increases. The surfactant molecules present in the flooding solution penetrate this interface and adsorb onto the rock surface. The contact angle increases towards the oil but decreases towards the surfactant liquid/rock. This change caused the oil to ripple and detach from the sandstone surface, forming an oil-in-water (O/W) emulsion. As a result, the amount of recovered oil increased. The following mechanism details the process of oil recovery by surfactant. The adsorption of surfactant molecules onto the rock surface is a physical process. Wettability alteration during surfactant flooding involves a series of physicochemical steps governed by surfactant adsorption, interfacial activity, and phase behavior.

1. Initial State:

Initially, crude oil strongly wets the rock surface, as evidenced by a high contact angle (θ >> 90°), indicating a strongly oil-wet system. This condition is stabilized by hydrophobic interactions between crude oil components and the mineral surface.

2. Pre-CMC Surfactant Addition:

At surfactant concentrations below the Critical Micelle Concentration (CMC), surfactant molecules exist primarily as monomers. In this region, adsorption at the oil–rock interface is limited and governed by weak van der Waals interactions. Although a slight reduction in contact angle is observed, the system remains predominantly oil-wet (θ > 90°). The adsorption behavior at this stage is strongly influenced by the hydrophilic–lipophilic balance (HLB), where insufficient surface coverage limits interfacial modification.

3. At the CMC:

At the CMC, surfactant molecules reach a critical packing density at the interface, forming a compact monolayer. This adsorption is driven by the amphiphilic nature of the molecules, where the hydrophobic tails anchor into the oil phase while the ethoxylated, hydrophilic heads interact with the aqueous phase. The molecular structure of the synthesized surfactants (aromatic EABS14 and aliphatic EHWO14) enhances interfacial packing efficiency through mixed micelle formation. At this stage, interfacial tension (IFT) decreases significantly due to disruption of cohesive oil–water interactions, while salinity plays a key role by compressing the electrical double layer, reducing electrostatic repulsion, and promoting tighter interfacial adsorption. Consequently, the contact angle decreases below 90°, indicating a transition toward a less oil-wet or intermediate-wet state.

4. Post-CMC Dynamics:

Beyond the CMC, the system evolves into a more stable microemulsion environment, where Winsor-type phase behavior (especially Winsor III near optimum salinity) enhances oil solubilization. The dynamic IFT continues to decrease due to continuous rearrangement of surfactant molecules at the interface. The synergistic interaction between EHWO14 and EABS14, along with the co-surfactant (isopropanol), improves HLB tuning and promotes the formation of flexible interfacial films. As a result, the contact angle further decreases (θ << 90°), and the rock surface becomes strongly water-wet due to near-complete coverage by an adsorbed surfactant monolayer.

5. Final Recovery Stage:

At optimum conditions, the system exhibits ultra-low interfacial tension and highly efficient wettability reversal. The crude oil viscosity is effectively reduced through emulsification, leading to the formation of stable oil-in-water (O/W) microemulsions. The synergistic surfactant blend (EHWO14 + EABS14 + co-surfactant) optimizes interfacial curvature and enhances solubilization capacity, resulting in maximum oil mobilization. The recovery factor reaches its highest value due to combined effects of ultra-low IFT, favorable wettability alteration, and improved phase behavior under optimal salinity conditions^51^.

The wettability state of the rock surface is commonly evaluated through contact angle (θ) measurements and the associated thermodynamic parameter, the work of adhesion (Wa).

The work of adhesion was calculated using the Young–Dupré equation:

5 $$Wa\,=\,\gamma{L}(1+cos\theta)$$

where γL is the oil–water interfacial tension (IFT) and θ is the measured contact angle. The calculated values are summarized in Table 3. The blank system exhibited a high contact angle (θ = 155°), indicating a strongly oil-wet surface with minimal adhesion between the aqueous phase and the rock. Upon addition of surfactants, a substantial reduction in contact angle was observed, confirming effective wettability alteration toward a water-wet condition. Among the tested systems, EHWO14 reduced the contact angle to 31°, while the ternary formulation (EHWO14 + EABS14+Cs) further decreased θ to 25°, indicating a stronger shift toward water-wetness. This behavior is attributed to the adsorption of surfactant molecules at the solid–liquid interface, forming a hydrophilic film that enhances the affinity of the rock surface toward the aqueous phase. The work of adhesion is governed by both interfacial tension and contact angle. While the ternary system exhibits ultra-low IFT, the dominant factor contributing to wettability alteration is the significant reduction in contact angle, which enhances fluid spreading and oil detachment. These findings confirm that wettability alteration, rather than IFT reduction alone, is a key mechanism driving improved oil recovery.

**Table 3:** Work Adhesion and Surface Free Energy of EHWO14 and its Blend.

Surfactant System	Spreading coefficient, Ws (–)	Work Adhesion, Wa (mJ/m²)	Contact Angle Ө (°)	Static IFT (mN/m)	Static ST (mN/m)
Blank	-3.72 × 10¹	1.83	155	19.5	67
EHWO14	-5.72 × 10⁻³	7.43 × 10⁻²	31	4 × 10⁻²	28.5
EHWO14 + EABS14	-2.34 × 10⁻²	1.77 × 10⁻¹	40	1 × 10⁻¹	25.2
EHWO14 + EABS14 + Cs	-9.40 × 10⁻⁴	1.91 × 10⁻²	25	1 × 10⁻²	21.8

### Surface charge energy

The relationship between interfacial tension, contact angle, and surface energies is described by Young’s equation:

6 $$\:{{\upgamma\:}}_{\mathrm{S}}={{\upgamma\:}}_{\mathrm{S}\mathrm{L}}+{{\upgamma\:}}_{\mathrm{L}}\mathrm{c}\mathrm{o}\mathrm{s}{\uptheta\:}$$

where $\:{\gamma\:}_{S}$is the solid surface free energy, $\:{\gamma\:}_{SL}$is the solid–liquid interfacial tension, and $\:{\gamma\:}_{L}$is the liquid surface/interfacial tension.

Accurate determination of $\:{\gamma\:}_{S}$requires independent measurement of $\:{\gamma\:}_{SL}$, which is not directly accessible in the present experimental framework. Therefore, quantitative evaluation of surface free energy was not pursued to avoid introducing significant uncertainty.

Instead, wettability alteration was assessed using directly measurable and reliable parameters, namely contact angle, interfacial tension, and work of adhesion. This approach ensures a robust interpretation of interfacial behavior without relying on assumptions that may compromise thermodynamic consistency.

The observed decrease in contact angle and IFT confirms enhanced solid–liquid interactions and supports the transition from an oil-wet to a water-wet system, which is essential for efficient oil displacement.

### Spreading coefficient of surfactant on the rock surface

The spreading coefficient (Ws) is a key thermodynamic parameter that describes the ability of a liquid phase to spread over a solid surface in the presence of another immiscible phase. It was calculated using:

7 $$\:{\mathrm{W}}_{\mathrm{s}}={{\upgamma\:}}_{\mathrm{L}}(\mathrm{c}\mathrm{o}\mathrm{s}{\uptheta\:}-1)$$

The calculated Ws values are presented in Table 3. For the blank system, the highly negative spreading coefficient reflects poor spreading of the aqueous phase over the oil-covered rock surface, consistent with its strongly oil-wet nature.

The introduction of surfactants significantly altered this behavior. EHWO14 reduced the magnitude of the negative spreading coefficient, while the ternary system (EHWO14 + EABS14+Cs) exhibited the least negative value. This trend indicates improved spreading of the aqueous phase and enhanced displacement of oil from the rock surface.

The improved spreading behavior of the ternary system can be attributed to the synergistic interaction between the nonionic surfactant, anionic surfactant, and co-surfactant. The presence of isopropanol as a co-surfactant enhances interfacial flexibility, promotes tighter molecular packing at the interface, and facilitates the formation of a stable oil-in-water (O/W) microemulsion. A less negative spreading coefficient, combined with ultra-low IFT and reduced contact angle, promotes the detachment of oil droplets and prevents their re-adhesion to the rock surface. This results in improved mobilization of trapped oil and higher recovery efficiency^52^.

### Enhanced oil recovery factor of the surfactant flooding

**Fig. 10:**
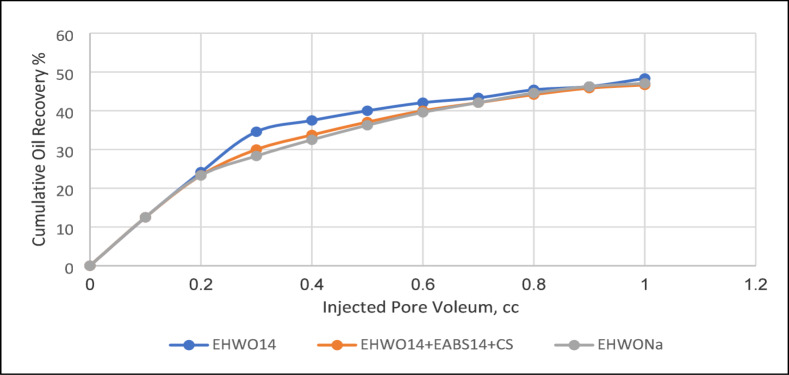
Secondary Recovery for EHWO14, EHWO14 + EABS14 + Cs, and. EHWONa at 50 ^◦^C and Salinity 100 × 10^3^ ppm.

**Fig. 11:**
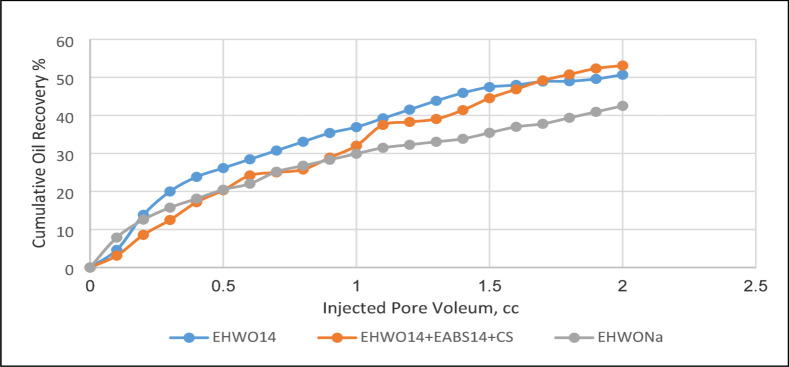
Tertiary Recovery for EHWO14, EHWO14 + EABS14 + Cs, and. EHWONa at 50 ^◦^C and Salinity 100 × 10^3^ ppm.

**Fig. 12:**
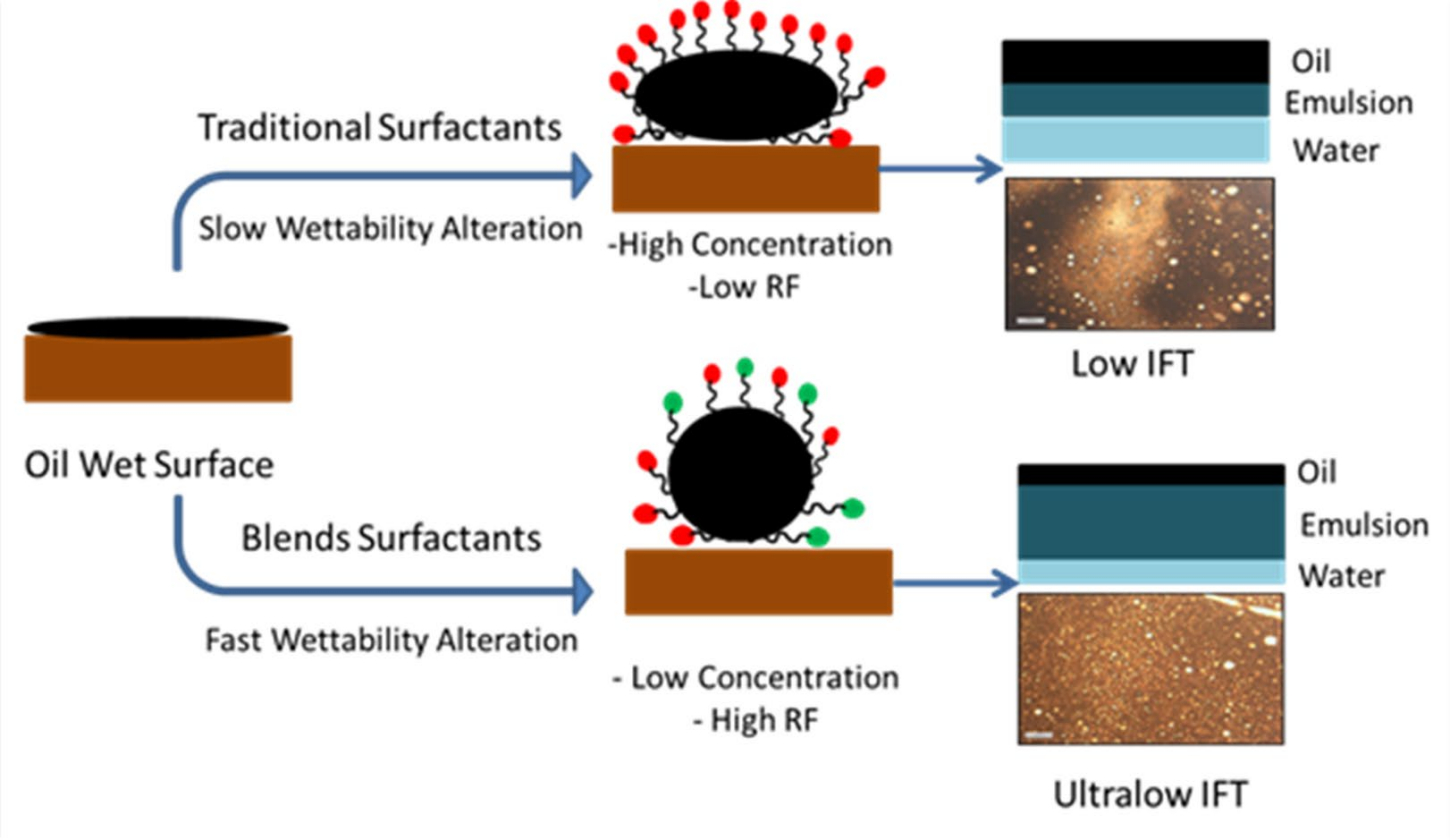
Molecular Mechanism of the Blends performance and Traditional Surfactants in EOR.

The key to surfactant flooding in enhanced oil recovery (EOR) is designing a surfactant slug with an optimal concentration. This process is influenced by various parameters, including temperature, critical micelle concentration (CMC), adsorption characteristics on the sand-packed model, interfacial tension, contact angle, and wettability alteration at the core-formation water interface. Flooding experiments were conducted for EHWO14 and its blends, both with and without the co-surfactant isopropanol. The experiments were performed at slightly above CMC concentrations, using different temperatures (50 °C, 70 °C) and salinities (50 × 10³, 100 × 10³, 200 × 10³ ppm) on a sand-packed model. The co-surfactant reduces the interfacial tension (IFT), which improves microemulsion stability by enhancing the solubility of surfactant molecules in the oil phase. This mechanism ultimately leads to a higher recovery factor (RF). As shown in Table 4, which presents the cumulative oil recovery percentages for EHWO14 and its blends, the maximum RF of 79% was achieved by the blend EHWO14 + EABS14+Cs at a salinity of 100 × 10³ ppm and a temperature of 50 °C. The action of surfactant molecules at the interface creates a complete adsorbed layer and a stable electric double layer. This leads to a reduction in interfacial tension (IFT) and, consequently, an increase in the oil recovery factor. To characterize this behavior, the solubilization parameters and phase behavior of the prepared surfactants were determined at different temperatures and salinities. The best performance, as shown in Figs. 10 and 11, was achieved by the surfactants and their blends at a salinity of 100 × 10³ ppm and a temperature of 50 °C. These results are attributed to the specific properties and behavior of the surfactants. Furthermore, maximum oil solubilization occurred at the optimal salinity and at concentrations above the critical micelle concentration (CMC). Conversely, recovery is low at concentrations both below and above the CMC. At concentrations significantly above the CMC, increased surfactant adsorption can form multilayers, leading to water-in-oil (W/O) emulsion that impedes oil recovery. The mechanism by which concentration affects the recovery factor was discussed in previous work^51^. Figure 12 illustrates a conceptual mechanism proposed in this study to explain the differences between individual surfactants and their blends. For surfactant blends, it is proposed that cooperative interactions between components facilitate the formation of more organized interfacial layers, which may enhance oil detachment from pore surfaces and favor the formation of oil-in-water (O/W) emulsions with lower apparent viscosity, thereby improving the recovery factor. In contrast, individual surfactants may exhibit less optimal interfacial organization under certain conditions, potentially leading to changes in emulsion behavior and reduced recovery efficiency. It should be emphasized that this mechanistic interpretation is speculative and intended to provide a qualitative framework for understanding the observed trends, pending further experimental confirmation.

**Table 4:** Cumulative Oil Recovery Percent at Different Salinities and Different Temperatures for EHWO14 and its Blends.

Subject	Salinity (ppm)	Temperature (°C)	Sec. Recovery		Ter. Recovery		Residual oil	Total Recovery	
			CC	%	CC	%	CC	CC	%
EHWO14	50 × 10³	50	58.00	48.33	21.75	35.08	62.00	79.75	66.45
EHWO14	100 × 10³	50	55.00	45.83	33.12	50.69	61.53	88.12	71.00
EHWO14	100 × 10³	70	54.19	47.12	26.00	42.75	60.81	80.19	66.82
EHWO14	200 × 10³	70	53.00	44.16	25.00	37.31	67.00	78	65.00
EABS14+EHWO14	200 × 10³	50	53.00	44.16	34.50	50.73	68.00	87.5	72.91
EABS14+EHWO14+Cs	100 × 10³	50	56.00	46.66	34.00	53.12	64.00	90	79.00
EHWONa	100 × 10³	50	56.00	48.69	32.00	54.23	59.00	88	73.33
EHWONa	100 × 10³	70	56.50	47.08	27.00	42.51	63.50	83.5	69.58
EHWONa+EHWO14	100 × 10³	50	58.50	48.75	29.00	47.15	61.50	87.5	72.91
EHWONa+EHWO14	100 × 10³	70	56.00	46.66	19.00	29.68	64.00	75	62.50

## Life-cycle environmental impact

A preliminary life-cycle perspective indicates that the use of waste cooking oil (WCO) as a feedstock substantially improves the environmental profile of the synthesized surfactants compared to conventional petrochemical analogues. Since WCO is a waste-derived raw material, its upstream environmental burden is minimal and can be reasonably treated as having near-zero or even negative allocation in life-cycle assessment (LCA) frameworks, depending on the adopted system boundaries. This significantly reduces cumulative energy demand (CED) and greenhouse gas (GHG) emissions associated with raw material extraction. The conversion of WCO into surfactants involves relatively mild reaction conditions (e.g., ethoxylation and sulfonation), which, although contributing to energy use and chemical inputs, remain less intensive than full petrochemical synthesis routes. Furthermore, the high efficiency of the formulated systems—demonstrated by ultra-low interfacial tension (down to 10⁻³ mN/m) and high oil recovery factors (up to 79%)—implies lower required dosages and reduced injection volumes, thereby decreasing chemical consumption per barrel of oil recovered. From a life-cycle impact standpoint, this translates into reduced emissions, lower water usage, and minimized environmental footprint during field application. Additionally, valorization of WCO mitigates environmental risks associated with its disposal, such as aquatic pollution and sewer blockage, contributing to improved end-of-life management. Overall, integrating WCO-derived surfactants into EOR operations aligns with circular economy principles and offers a favorable balance between process performance, environmental impact, and economic feasibility.

## Conclusions

- This study examined the relationship between the molecular characteristics of green surfactants derived from waste cooking oil and their interfacial behavior, microemulsion phase behavior, and enhanced oil recovery (EOR) performance using a sand-pack flooding model. The main findings are summarized as follows:
- The synthesized surfactants (nonionic, anionic, and blended systems) exhibited distinct phase behavior depending on their molecular structure and formulation. These variations influenced microemulsion formation, solubilization capacity, and interfacial activity under different salinity and temperature conditions.
- The incorporation of a co-surfactant (Cs) in the EHWO14 + EABS14 + Cs system significantly improved interfacial performance. At 50 °C and an optimum salinity of 100 × 10³ ppm, the interfacial tension (IFT) decreased to 1 × 10⁻³ mN/m, compared with 1 × 10⁻² mN/m for EHWO14 and 8 × 10⁻² mN/m for the EHWO14 + EABS14 blend, indicating a synergistic effect of the formulation.
- Wettability measurements showed a reduction in contact angle from 159° to 22° for the most effective formulation, compared to 27° and 35° for EHWO14 and EHWO14 + EABS14, respectively, suggesting a shift toward more water-wet conditions.
- Phase behavior analysis confirmed that optimum microemulsion formation and minimum IFT occurred at 100 × 10³ ppm salinity, highlighting the importance of salinity in controlling surfactant phase behavior.
- Microscopic observations indicated that anionic surfactants enhanced oil-in-water emulsion formation, while nonionic-based blends contributed to improved emulsion stability, both of which support oil mobilization.
- Interfacial parameters (work of adhesion, surface free energy, and spreading coefficient) indicated favorable conditions for oil detachment from the rock surface and subsequent transport as emulsified droplets.
- Sand-pack flooding experiments showed total oil recoveries of 71% (EHWO14), 72.91% (EHWO14 + EABS14), and 69.58% (EHWONa) under the studied conditions, demonstrating comparable performance among the tested systems.
- Overall, the results indicate that surfactant molecular structure, co-surfactant addition, and salinity collectively govern interfacial behavior and phase characteristics, which in turn influence EOR performance. These findings provide a consistent experimental basis for understanding structure–property–performance relationships in bio-based surfactant systems for potential EOR applications.

## Supplementary Information

Below is the link to the electronic supplementary material.

Supplementary Material 1

## Data Availability

The raw datasets used and/or analyzed during the current study are available from the corresponding author on reasonable request.
