# Supplementary figures and images for: Conversion of waste cooking oil to environmentally acceptable surfactants for enhanced oil recovery
Source: Sci Rep. 2026 Jun 6;16:17535. doi: 10.1038/s41598-026-55003-z (PMC13242505; doi:10.1038/s41598-026-55003-z)

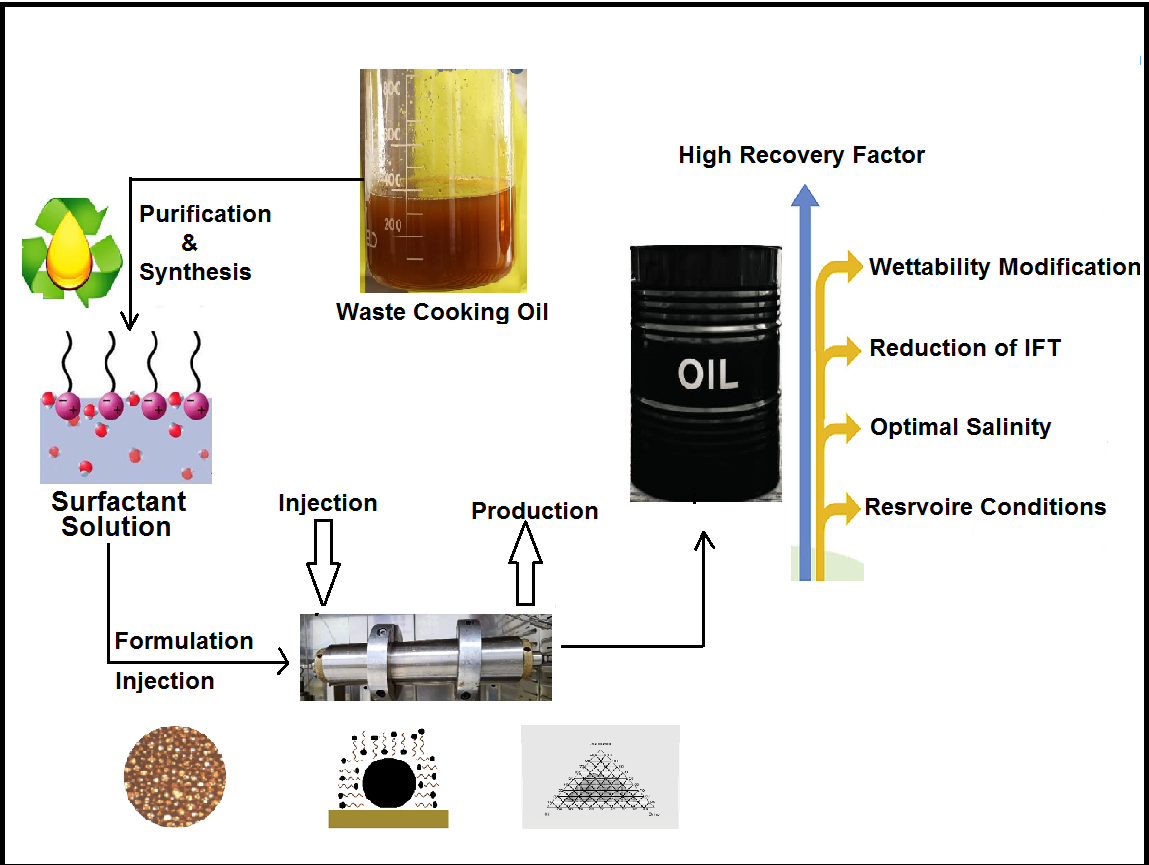

Supplement: Supplementary file 1 — Supplementary Material 1 [file 41598_2026_55003_MOESM1_ESM.docx]
